# Massive lithospheric delamination in southeastern Tibet facilitating continental extrusion

**DOI:** 10.1093/nsr/nwab174

**Published:** 2021-09-13

**Authors:** Jikun Feng, Huajian Yao, Ling Chen, Weitao Wang

**Affiliations:** Laboratory of Seismology and Physics of Earth's Interior, School of Earth and Space Sciences, University of Science and Technology of China, Hefei 230026, China; Laboratory of Seismology and Physics of Earth's Interior, School of Earth and Space Sciences, University of Science and Technology of China, Hefei 230026, China; CAS Center for Excellence in Comparative Planetology, University of Science and Technology of China, Hefei 230026, China; Mengcheng National Geophysical Observatory, University of Science and Technology of China, Mengcheng 253500, China; State Key Laboratory of Lithospheric Evolution, Institute of Geology and Geophysics, Chinese Academy of Sciences, Beijing 100029, China; CAS Center for Excellence in Deep Earth Science, Guangzhou 510640, China; Institute of Geophysics, China Earthquake Administration, Beijing 100081, China

**Keywords:** ambient noise interferometry, reflected body waves, lithospheric delamination, India-Asia collision, Indochina extrusion

## Abstract

Significant left-lateral movement along the Ailao Shan-Red River fault accommodated a substantial amount of the late Eocene to early Miocene India-Asia convergence. However, the activation of this critical strike-slip fault remains poorly understood. Here, we show key seismic evidence for the occurrence of massive lithospheric delamination in southeastern Tibet. Our novel observation of reflected body waves (e.g. *P_410_P* and *P_660_P*) retrieved from ambient noise interferometry sheds new light on the massive foundered lithosphere currently near the bottom of the mantle transition zone beneath southeastern Tibet. By integrating the novel seismic and pre-existing geochemical observations, we highlight a linkage between massive lithospheric delamination shortly after the onset of hard collision and activation of continental extrusion along the Ailao Shan-Red River fault. This information provides critical insight into the early-stage evolution of the India-Asia collision in southeastern Tibet, which has significant implications for continental collision and its intracontinental response.

## Teaser

New findings of the delaminated continental lithosphere at the bottom mantle transition zone beneath southeastern Tibet provides new insights into the continental extrusion process of the Tibetan Plateau.

## INTRODUCTION

The continuous convergence between India and Asia since ∼60 Ma has raised the spectacular Tibetan Plateau and Himalayan mountain belt [1–3], which provide an important natural laboratory to study the details of continental collision. Since the onset of continental collision, at least 1500 km of intracontinental convergence has been accommodated by lithospheric thickening, continental extrusion outside the plateau and lithospheric foundering into the deep mantle [1,4]. Recent geodetic observations based on the global positioning system (GPS) indicate vast internal east-west extension within the plateau and significant eastward continental extrusion outside the plateau [5,6]. Apparently, the eastward extrusion is modulated by the variations in mechanical strength along the peripheral lithosphere, and southeastern Tibet provides a weakened window for the most significant material ‘escape’ from the Tibetan Plateau [5,7]. The lithosphere beneath southeastern Tibet is thin [8,9], underlain by significant low-velocity anomalies [10] and accommodates significant shear strains [5]. However, little is known about the formation of such a weakened window.

Significant lithospheric deformation, including rotation and extrusion, has occurred within southeastern Tibet since the Cenozoic [11–13]. The Indochina block was extruded to the southeast for 700 ± 200 km along the Ailao Shan-Red River fault (ASRRF) that cuts through the entire lithosphere [13,14]. How a rigid continental block could be extruded from the southeastern margin of the Tibetan Plateau is fundamental for understanding the regional tectonic process and the rheological structure of the lithosphere. Lithospheric delamination and bottom heating beneath southeastern Tibet have been postulated to explain the Cenozoic potassium-rich magmas in Southwest China [15–17], analogous to the scenario in the central plateau [18–20]. However, conclusive seismic evidence for the foundered lithosphere in the deep mantle is lacking. Recent progress in ambient noise analysis and imaging provides us with new opportunities to study the Earth's internal structure from a unique perspective without earthquakes [21–23]. Here, we try to shed new light on the deep mantle structure and shallow tectonic evolution beneath southeastern Tibet based on the novel observation of reflected body waves retrieved from ambient noise interferometry.

## RESULTS

### Retrieval of reflected body waves

**Figure 1.**
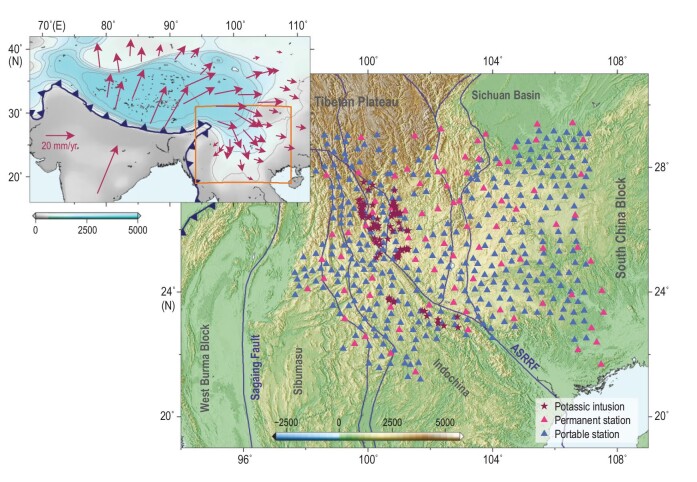
Station map and geological setting. Red and blue triangles represent permanent and portable seismic stations, respectively. Dark red stars denote the locations of Eocene-Oligocene potassic intrusions (from ref. [17] and references therein). Thin blue lines indicate major tectonic boundaries. ASRRF: Ailao Shan-Red River fault. The upper-left inset exhibits selected GPS velocities with respect to a stable Eurasia from ref. [5]. The background depicts smoothed topography. The dark blue line shows the suture zone. The orange rectangle denotes the study region.

Despite the fact that the ambient noise interferometry technique has been widely applied to explore the Earth's internal structure by retrieving body wave phases [23,24], most of these body wave phases retrieved from ambient noise cross-correlation functions (NCFs) present poor signal-to-noise ratios. Here, we combined the seismic observations from a dense seismic array and the phase-weighted stacking method for common reflection points to improve the signal-to-noise ratio of deep reflected body waves [21,25]. We first collected continuous vertical component seismograms recorded by 350 portable and 88 permanent seismometers from March 2011 to November 2013 in Southwest China (Fig. 1). Then, NCFs were calculated between all available station pairs and filtered to 0.1–0.2 Hz (Fig. S2). To probe the deep mantle structure and potential scatters, we performed phase-weighted stacking for the common reflection points of 95 633 NCFs to retrieve reflected body waves (*P_410_P*, *P_660_P* and potential scattered signals from other depths) [21].

**Figure 2.**
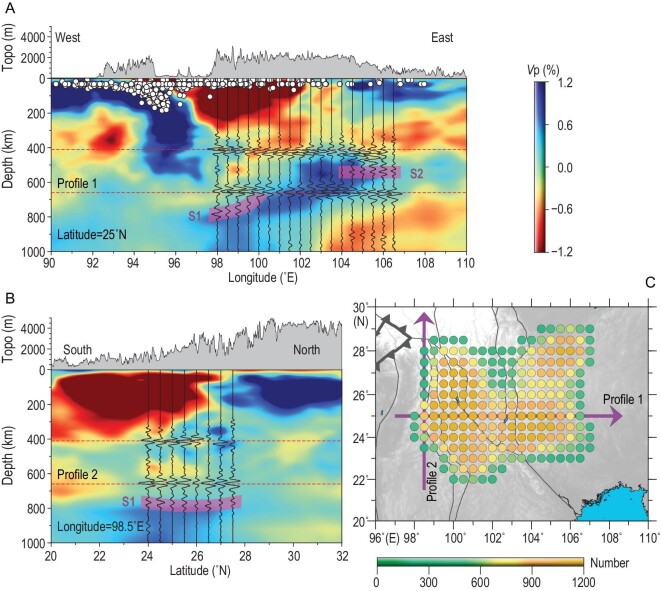
Cross sections of reflected waves and data density. (A) and (B) Cross sections of stacked depth-domain NCF waveforms labeled Profile 1 and Profile 2 in (C), respectively. The shallow part (<300 km) of the depth-domain traces is suppressed with a taper. The background shows the P-wave velocity perturbation calculated from the UU-P07 model [27]. White circles represent the seismicity (M > 4) along Profile 1. (C) Locations of common reflection point stacking bin centers, with the color representing the number of stacked NCFs within each bin. Here, one bin is selected only if the number of stacked NCFs is >300.

To account for the difference in interstation spacing, all time-domain NCFs, regarded as reflection signals from different depths, were converted to depth-domain NCFs based on the 1D iasp91 model [26]. Following our previous study [21], the depth-domain NCFs were stacked with a phase-weighted stacking method for common reflection points (Fig. 2). Here, a circle with a radius of 1° was set as the bin for common reflection point stacking. To avoid the interference from energetic surface waves (Fig. S2), only the NCFs whose offsets were less than 200 km were adopted in the stacking. The locations of the bin centers and the corresponding numbers of stacked NCFs are shown in Fig. 2C.

Reflected body waves from the 410- and 660-km discontinuities can be observed clearly on cross sections of stacked depth-domain NCFs (Fig. 2A and B). In addition to the major reflected phases *P_410_P* and *P_660_P*, distinct signals S1 and S2 scattered in the uppermost lower mantle and the middle mantle transition zone (MTZ) emerge on the cross sections. Our observations show striking agreement with the UU-P07 model [27] (Fig. 2A and B) and the MIT08 model [28] (Fig. S5), indicating a west-dipping high-velocity anomaly penetrating the 660-km interface. The distinct signal S1 clearly corresponds to the reflection from the upper boundary of the high-velocity anomaly near its western end. The signal S2 is spatially related to the eastern end of the high-velocity anomaly within the MTZ. One attractive mechanism for S2 is the upside reflection from the heterogeneities generated by the interaction of the foundered lithosphere with the surrounding mantle. Although UU-P07 and MIT08 are global models with limited resolution, their major features are compatible with our high-resolution imaging results.

### Topography of mantle transition zone discontinuities

**Figure 3.**
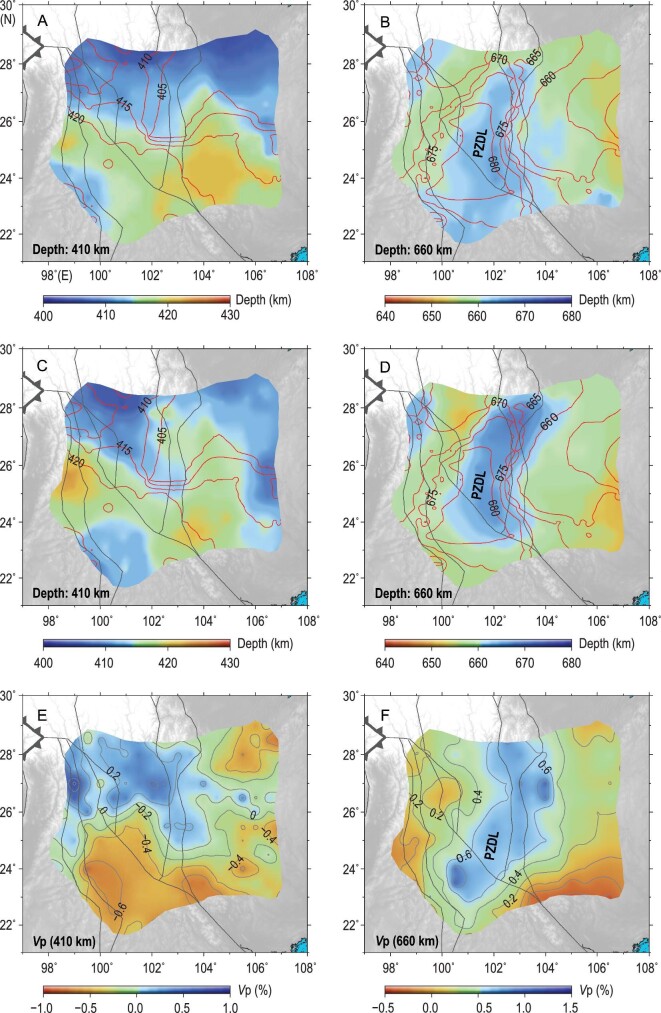
Mantle discontinuity depths and seismic velocity structures. (A) and (B) The color represents the topography of the 410- and 660-km discontinuities, respectively, estimated from ambient noise interferometry data based on the 1D iasp91 model. (C) and (D) The topography of mantle discontinuities estimated using the 3D FWEA18 model [30]. The red contours in (A–D) depict the mantle discontinuity depths from conventional receiver functions [29] based on the 1D iasp91 model. (E) and (F) P-wave velocity perturbations (UU-P07 model [27]) at depths of 410 and 660 km, respectively. PZDL: penetration zone of the delaminated lithosphere. Thin black lines denote the major tectonic boundaries.

To determine the relative topography of the 410- and 660-km discontinuities, we measured the depth difference between all neighboring traces by calculating the cross correlation of the target reflection phase. Therefore, the spatial topographic variations in mantle discontinuities are much more accurate than their absolute depths. As the NCFs were first converted to depth domain based on the 1D iasp91 model without taking account of the 3D velocity structure, the topographies (Fig. 3A and B) actually represent the joint effect of the real discontinuity topography and the overlying seismic velocity structure. To confirm the reliability of this novel method, the topographies estimated from ambient noise interferometry were compared with those from conventional receiver functions [29], which were also based on the 1D iasp91 model. Though these two methods were most sensitive to *Vp* and *Vs*, respectively, the straightforward comparison shown in Fig. 3A and B and Fig. S6, based on a 1D model, excludes the influence of complex 3D *Vp* and *Vs* structure. The consistency between the different results intuitively proves the effectiveness of this novel method of measuring the topography of the mantle discontinuities.

To take the effect of 3D *Vp* structure into account, a regional seismic velocity model, FWEA18 [30], constructed using full waveform inversion, was further adopted to determine the discontinuity depths (Fig. 3C and D). The FWEA18 model was adopted mainly because it provides the absolute seismic velocity values rather than velocity perturbation, which is obtained by teleseismic traveltime tomography. The topography of the mantle discontinuities was also compared with the tomography slices at corresponding depths (Fig. 3E and F). These three independent observations show good consistency, further demonstrating the reliability of the reflected body waves retrieved from ambient noise interferometry.

The most striking feature of the 410-km discontinuity is the north–south contrast in topography. The 410-km discontinuity is shallower than the regional average 410-km discontinuity depth north of 26°N, especially the northwestern part of the study region, where higher wave velocities (cold) dominate. By contrast, the 410-km discontinuity is deeper than the regional average 410-km discontinuity depth south of 26°N, where lower wave velocities (hot) are predominant. These observations can be simply reconciled by a positive Clapeyron slope, which is expected for the phase transition from olivine to wadsleyite around a 410-km depth [31,32]. On the other hand, the 660-km discontinuity exhibits a remarkable depression between 100°E and 103°E, an area characterized by the highest wave velocities within the study region due to the penetration of a high-velocity anomaly to the lower mantle (Figs 2A and 3F). These observations at the bottom of the MTZ indicate a negative Clapeyron slope, which is expected for the phase transition from ringwoodite to bridgmanite and magnesiowüstite around a 660-km depth [31,32]. In short, the topography of the mantle discontinuities is mainly controlled by lateral temperature variations. This is compatible with the results of mineralogical experiments [33,34].

## DISCUSSION

By integrating reflected waveforms, the topography of the mantle discontinuities [29,35] and previous tomography models [27,28], we propose the existence of a massive west-dipping high-velocity anomaly sinking from the MTZ to the lower mantle. Although high-velocity anomalies near the MTZ have long been observed beneath southeastern Tibet [36,37], there is still no consensus on the origin of such anomalies, mainly due to the lack of fine constraints on their spatial features.

**Figure 4.**
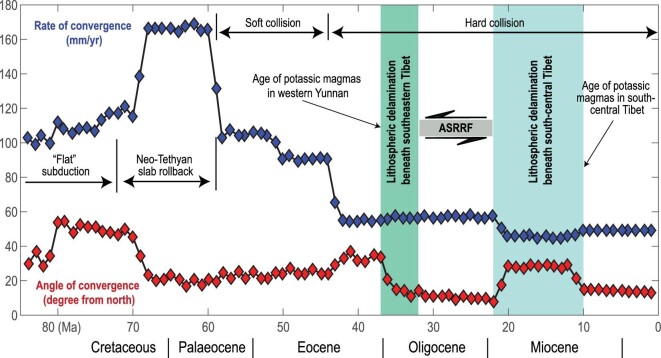
India-Asia convergence and major tectonic events. Both lithospheric delamination events, occurring beneath southeastern and south-central Tibet, remarkably influenced the India-Asia collision. The occurrence of lithospheric delamination beneath southeastern Tibet occurred shortly after the onset of ‘hard’ collision, which in turn facilitated the initiation of sinistral shearing along the ASRRF. The red and blue diamonds represent the angle and rate of convergence between India and Eurasia, respectively. This figure was compiled from refs [3] and [41].

High-velocity anomalies within the MTZ were interpreted as the eastward subducted Indian slab [36,38]. However, several features are incompatible with such a scenario. First, the high-velocity anomaly within the MTZ is not connected with the subducting Indian plate in the shallow upper mantle (Fig. 2A and Fig. S5A), and the location of the high-velocity anomaly is not consistent with the reconstructed subduction zone at the point when the Indian ocean slab was detached from the continental plate. The detachment of the oceanic slabs from the Indian continent was proposed to take place at ∼45 Ma [39–41], manifested by a sudden drop in convergence rate (Fig. 4), and the submerged oceanic slab was imaged in the lower mantle, >1000 km south of the study region [42,43]. The imaged oceanic slab is consistent with the reconstructed subduction zones when the hard collision between India and Eurasia initiated (Fig. S7). Second, the eastward subducted Indian plate is more likely to produce an east-dipping high-velocity anomaly near the bottom MTZ east of the subduction zone [28,44], which is contrary to our observation and previous results (Fig. 2A and Fig. S8). Moreover, the narrow depression of the 660-km interface is unlikely to be caused by a stagnant slab due to the eastward subduction of the Indian plate beneath Southwest China. A flattened slab extending eastward for >1000 km within the MTZ should cause widespread depression of the 660-km interface rather than a narrow north–south band. Therefore, the high-velocity anomaly within the MTZ is unlikely to be the detached India slab, which should have already sunk into the lower mantle during the continuous northeastward movement of the Indian plate [42,43]. The high-velocity anomaly in the MTZ was previously interpreted as the subducted Indian slab mainly because the regional models usually fail to portray the intact morphology of this anomaly.

**Figure 5.**
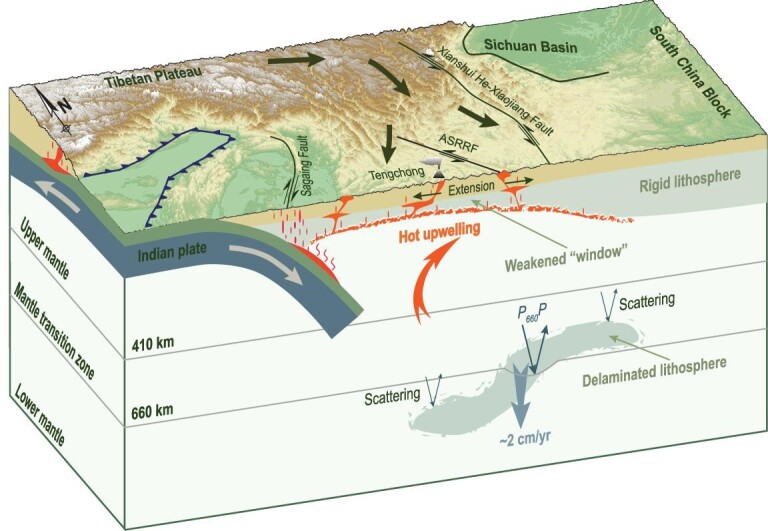
Lithospheric delamination facilitates continental extrusion. This figure shows a comprehensive interpretation by integrating tectonics, and geochemical and geophysical (including the present study) observations. Massive delamination of the continental lithosphere occurred beneath southeastern Tibet, inducing significant upwelling of the hot asthenosphere. Lithospheric thinning and bottom heating further led to partial melting of the remnant lithosphere (the red material beneath and within the remnant lithosphere) and potassic magmatism [16,17]. All these processes significantly weakened the lithosphere, which facilitated the initiation of the ASRRF and continental extrusion away from Tibet. Black arrows indicate the direction of material extrusion from central Tibet. The red arrow represents the upwelling of the hot asthenosphere. The initiation of the left-lateral shearing along the ASRR postdates the emplacement of the potassic magmatism [16,49].

An alternative origin of such a high-velocity anomaly is massive lithospheric delamination during ∼30–40 Ma (Fig. 5), several million years after the onset of a ‘hard’ collision between India and Asia [3,15,17]. The detachment of the oceanic slab from the Indian continent initiated the hard collision at ∼45 Ma, and is signified by sudden changes in the convergence rate and angle [3,41] (Fig. 4). The hard collision may have further thickened the continental lithosphere beneath southeastern Tibet and caused massive delamination of the thickened lithospheric mantle just a few million years later [45,46]. Massive lithospheric delamination inevitably leads to the upwelling of the hot asthenosphere and bottom heating of the thinned lithosphere (Fig. 5), which may further result in partial melting of the remnant lithosphere and associated magmatism [15–17]. The P-wave tomography model shows strong agreement with such a scenario by revealing a gap in the high-velocity lithosphere and the presence of distinct low-velocity upwelling above the delaminated high-velocity lithosphere (Fig. S9). A thin lithosphere, estimated from receiver functions [8,9], and significant low velocity at shallow depths (<100 km), revealed by high-resolution surface wave tomography [10], further support our model. However, no significantly thinned (low-speed) lithosphere was observed above the delaminated lithosphere in the northern part of the study area (profile 5 and 6 in Fig. S4). This is plausible because the shallow lithosphere within the study region moved southward after the lithospheric delamination, which can be inferred from the GPS velocity map (Fig. 1). Thus, the shallow thinned lithosphere and the deep delaminated lithosphere could have been situated in different locations. Moreover, the northern part of the study area, near the eastern Himalayan syntaxis, has experienced intense tectonic compression, which may significantly shorten the low-velocity window between the subduction zone and the South China Block.

The late Eocene to early Oligocene lithospheric delamination also corresponds to a sudden change in convergence angle without notable variation in convergence rate (Fig. 4) [3], which implies a counterclockwise rotation of the Indian plate. Such a counterclockwise rotation was plausibly induced by a sudden decrease in resistance resulting from massive lithospheric delamination and resultant lithospheric thinning and weakening in southeastern Tibet. This lithospheric thinning and weakening further facilitated the sinistral displacement along the ASRRF, which was estimated as being from 32 Ma to 22 Ma by recent thermochronological studies (Fig. 4) [47–49]. Lithospheric delamination has also been widely discussed with regard to the central Tibetan Plateau to explain the current topography and the underthrusting of the Indian plate [18,19,41]. The coincidence of both lithospheric delamination events with notable changes in convergence angle and/or rate indicates that these two events have profoundly influenced the continental collision (Fig. 4).

This scenario is also corroborated by the late Palaeogene high-potassium magmas in southeasten Tibet [15–17] (Figs 1 and 4). These high-potassium rocks were emplaced over a 200-km-wide zone across the ASRRF and are unlikely to be genetically related to shearing [16]. In comparison, the late Miocene to Quaternary potassium-rich magmas in central Tibet are attributed to lithospheric delamination and associated hot asthenospheric upwelling [18,20]. Similarly, voluminous Eocene-Oligocene volcanic potassic-ultrapotassic rocks were also reported in the central Tibetan Plateau [50,51]. An analogous dynamic model involving lithospheric delamination and partial melting of the remnant lithosphere was proposed to generate these volcanic potassic-ultrapotassic rocks [17,50]. Sudden changes in the thermal structure of the remnant lithosphere induced by lithospheric delamination may cause partial melting and potassium-rich magmatism (Fig. 5) [16,17]. Therefore, the ages and distribution of the high-potassium rocks provide primary constraints on lithospheric delamination. The high-potassium rocks probably indicate the presence of a weak lithosphere, where lithospheric shearing is more likely to take place as a result of continental extrusion. The best estimated age of the left-lateral shearing along the ASRR shear zone postdates the emplacement of the potassic magmas [16,48,49]. Therefore, these high-potassium rocks in southeastern Tibet are more likely related to massive continental lithospheric delamination and partial melting of the remnant lithosphere before the initiation of shearing along the ASRRF (Figs 4 and 5).

The high-velocity foundered lithosphere has already sunk into the MTZ and partly penetrated the lower mantle (Fig. 2A and B, and Fig. S8). The scattered signal S1 and S2 correspond to the west and the east margin of the west-dipping high-velocity anomaly, respectively (Fig. S8). Though the location of scattered signals does not perfectly coincide with the high velocity within the MTZ, mainly due to the limited resolution of the global models (Figs S4 and S5), a first-order spatial correspondence between the scatter waves and the high-velocity anomaly could be confirmed by the statistical vote maps (Fig. S8). The narrow depression zone of the 660-km interface is more likely induced by the penetration of the foundered lithosphere than by the stagnant Indian slab [35]. Considering the age span of the high-potassium magmas in western Yunnan (∼32–37 Ma) [16,17,46] and the depth range of the foundered lithosphere (∼600–800 km), the sinking rate of the delaminated continental lithosphere is estimated to be ∼2 ± 0.5 cm/year, which is slightly smaller than the free sinking rate of an oceanic slab in the upper mantle [43,52] mainly because of the difference in density. The sinking rate may also be underestimated due to short stagnation at the bottom of the MTZ. Obviously, the resistance from the 660-km interface did not stop the foundered lithosphere from sinking further into the lower mantle. The occurrence of massive lithosphere delamination is much earlier beneath southeasten Tibet than it is beneath the central Tibetan Plateau according to the age of high-potassium rocks in two regions [41] (Fig. 4). The foundering lithosphere is imaged mainly in the upper mantle beneath the south-central Tibet Plateau [19].

Although widespread lithospheric delamination has been proposed to account for mass balance during intracontinental convergence, relative to pronounced crustal shortening and continental extrusion the consumption of mantle lithosphere is much more enigmatic [19,41,53]. This study provides new seismic evidence, with the novel ambient noise interferometry technique, for the occurrence of massive lithospheric delamination beneath southeastern Tibet before the rapid southeastward extrusion of the Indochina Block. The delamination was probably triggered by the hard collision between India and Asia and subsequent lithospheric thickening [45,53]. As a corollary, there would have been more intense compressional deformation in southeastern Tibet than previously expected since the onset of the hard collision. Even if continental extrusion is regarded as an inevitable response to the India-Asia collision [54], it is difficult to shear massive rigid continental blocks without any weakening in advance. The massive lithospheric delamination and weakening may have significantly facilitated the initiation of the ASRRF and thus continental extrusion in the late Eocene–early Oligocene (Fig. 5). At present, this weakened window is still playing an important role in accommodating intracontinental convergence [5,7,55]. These details provide new insights into the early stage of the continental collision between India and Asia.

To the best of our knowledge, this study provides the first detailed comparisons between the 3D topographies of the MTZ discontinuities estimated from NCFs and receiver functions, and further demonstrates the accuracy of reflected body waves retrieved from ambient noise interferometry. These reflected body waves retrieved from NCFs have distinct sensitivities to the Earth's structure compared to conventional receiver functions, and higher resolution than PP and SS precursors. This novel method creates a new opportunity for probing the Earth's deep interior without earthquakes.

## MATERIALS AND METHODS

We adopted the upside reflected body waves (e.g. *P_410_P*, *P_660_P* and potential scattered signals from other depths) retrieved from 95 633 NCFs to image the MTZ discontinuities and potential large-scale scatters beneath southeastern Tibet. Figure S1 shows the ray geometries of *P_410_P* and *P_660_P*, the sampling region and general geological settings. The reflected body waves were employed to image deep mantle structures and further integrated with pre-existing multidisciplinary data to reveal massive lithospheric delamination beneath southeastern Tibet. More details are given in the supplementary data file.

## DATA AVAILABILITY

The continuous seismograms recorded by the permanent stations are archived at the Data Management Centre of the China National Seismic Network at the Institute of Geophysics, China Earthquake Administration (doi:10.11998/SeisDmc/SN, http://www.seisdmc.ac.cn). The continuous waveforms of portable stations were achieved at the China Seismic Array Data Management Centre at the Institute of Geophysics, China Earthquake Administration (doi:10.12001/ChinArray.Data, http://www.chinarraydmc.cn). The digital elevation data used to generate maps are publicly available at https://topex.ucsd.edu/WWW_html/srtm30_plus.html. The earthquake catalog used in this study was downloaded from https://earthquake.usgs.gov/earthquakes/search/. The codes to perform ambient noise analysis are available from the corresponding author. Most figures were generated with Generic Mapping Tools (GMT) (https://www.generic-mapping-tools.org).

## Supplementary Material

nwab174_Supplemental_File
