# Supplementary material for: Massive lithospheric delamination in southeastern Tibet facilitating continental extrusion
Source: Natl Sci Rev. 2021 Sep 13;9(4):nwab174. doi: 10.1093/nsr/nwab174 (PMC8982193; doi:10.1093/nsr/nwab174)
Supplement: nwab174_Supplemental_File [file nwab174_supplemental_file.docx]

Supplementary Materials for

**Massive lithospheric delamination in southeastern Tibet facilitating continental extrusion**

Jikun Feng^1*^, Huajian Yao^1,2,3^^*^, Ling Chen^4,5^, Weitao Wang^6^

*Corresponding author. Email: Jikun Feng ([jkfeng@mail.ustc.edu.cn](mailto:jkfeng@mail.ustc.edu.cn)) and Huajian Yao ([hjyao@ustc.edu.cn](mailto:hjyao@ustc.edu.cn))

**This PDF file includes:**

Supplementary Materials and Methods

Figs. S1 to S9

Supplementary References

**Supplementary Materials and Methods**

**Ambient noise cross-correlation calculation.** The routine ambient noise procedure presented in ref(*1*) was followed here to calculate the NCFs. The detailed procedure of single-station data pre-processing and ambient noise cross-correlation calculation can be found in ref(*2*). During ambient noise analysis, the continuous seismograms were down-sampled to one sample per second before final cross-correlation. Finally, 95,633 NCFs were available for further analysis in this study.

To check the reflected body wave signals in the NCFs, all the NCFs were filtered to the secondary microseism band ranging from 0.1 to 0.2 Hz and arranged with respect to the interstation distance (Fig. S2). Clear body wave signals (*P_410_P* and *P**_660_P*) reflected from the MTZ discontinuities and dominant Rayleigh waves emerge on the NCFs section. And the reflected phases show good alignment with respect to the theoretical traveltime curves. To further determine the ray parameters of the reflected body waves, all the time-domain NCFs were converted to the time-slowness domain by the slant stacking method (*3*). Slant stacking was performed within four distance ranges (50-250 km, 150-350 km, 250-450 km, and 350-550 km). Within each distance range, the arrival times and ray parameters of the reflected body waves that emerged in the NCFs show perfect agreement with the corresponding theoretical values of *P_410_P* and *P_660_P* calculated from the iasp91 model (*4*) with the TauP Toolkit software (*5*) (Fig. S3). We can conclude that NCFs can be applied to retrieve reliable body wave signals reflected from the mantle transition zone discontinuities.

**Phase-weighted stacking for common reflection points.** To account for the differences in interstation distances, all the time-domain NCFs were first converted to the depth domain based on the 1-D iasp91 model. When performing the time-to-depth conversion, each point of the time-domain waveform was regarded as a reflection signal from the deep Earth, which was not suitable for the shallow part due to the interferences from energetic surface waves. Therefore, the shallow part (<300 km) of depth-domain traces was suppressed with a taper.

Then, a series of stacking bins were defined with a circle with a radius of 1°. The bin centers were set on the grid nodes of a 0.5° by 0.5° network within the study region (98°E-107°E; 22°N-29°N), as shown in Fig. 2C. For each bin, the depth-domain NCFs whose reflection points were located within the corresponding bin were collected and stacked with a phase-weighted stacking method (*6, 7*). Repeating the above process for all the bins, we obtained all the stacked depth-domain reflected waveforms (Fig. 2A, 2B, and Fig. S4). Clear *P_410_P* and *P_660_P* phases, defining the top and bottom interfaces of the MTZ, can be observed on the cross-sections. Significant waveform changes in *P_660_P* can also be observed where the high-velocity anomaly penetrates to the lower mantle. In addition to the major *P_410_P* and *P_660_P* phases, distinct signals (S1 and S2 in Fig. 2) corresponding to the west and east ends of the high-velocity anomaly also emerge on the cross-sections.

**Topography of** **the 410- and 660-km discontinuities.** The relative topographies of the 410- and 660-km discontinuities were estimated by calculating the cross-correlation of the target phases from all neighboring bins. First, two depth-domain windows (360-460 km and 610-710 km) were designed to isolate the waveforms of *P_410_P* and *P_660_P*, respectively. Then, the depth differences of the mantle discontinuity (the 410- or the 660-km discontinuity) between any two neighboring bins were measured by calculating the cross-correlation of the corresponding isolated waveforms. The reliability of the measured depth differences was judged by the maximum of the cross-correlations. A measurement was accepted only when the maximum of the corresponding cross-correlation was larger than 0.85. Repeating the above process, we obtained the depth differences of the mantle discontinuities between any two adjacent bins. When a reference depth of the 410-km discontinuity (or 660-km discontinuity) was assigned to one central bin, the relative depths of the surrounding bins were easily obtained by considering the depth differences. Intuitively, the relative depth measurement continues expanding outward until it reaches the boundary. The topographies of the 410- and 660-km discontinuities measured from ambient noise interferometry data show good consistency with those from conventional receiver functions (Fig. 3A, 3B, and S6).

As the upside reflected P-waves (*P_410_P* and *P_660_P*) and receiver functions have distinct sensitivities to the P-wave and S-wave velocity models, the discrepancy between the P-wave and S-wave models may be further transferred to the final topography. For a more intuitive comparison, the calculations of the mantle interface depths from both ambient noise interferometry and conventional receiver functions are based on the iasp91 model. Although the influence of the 3-D model is not removed, the major features of the mantle discontinuity topographies estimated by different methods show great agreement. The results from receiver function show lager topographic variation than those from ambient noise interferometry mainly because of the spatial smoothing induced by common reflection point stacking (Fig. S6). The size of the common reflection stacking bin is set to 1 degree. There is also a good correspondence between the mantle discontinuity topographies and the 3-D P-wave velocity model (Fig. 3).

**Calculation of high-velocity vote maps**. To further confirm the existence of a west-dipping high-velocity anomaly near the bottom MTZ, eight recognized P-wave velocity models (DETOX-P3 (*8*), GAP-P4 (*9*), Hosseini2016, MIT08 (*10*), MIT_USA_2011MAR (*11*), MIT_USA_2016MAY (*12*), TX2019slab-P (*13*), and UU-P07 (*14*) provided by SubMachine (*15*)) were analysis with SubMachine to generate the high-velocity vote maps above and beneath the 660-km interfaces following ref(*16*) (Fig. S8). Tomography vote maps, indicating the regions of agreement and disagreement, were calculated from stacking depth slices of several tomography models. First, a binary threshold of 0 or 1 was determined for each grid point (pixel) of every depth slice. For a high-velocity vote map, a value of 1 (“yes”) was applied to the pixels where the perturbations of seismic velocities were larger than the standard deviation of the tomography model at given depth. Otherwise, the pixels were set to 0 (“no”). Then, all the binary depth slices were summed into a vote map (Fig. S8). Hence, each pixel of a vote map takes a value between 0 and the total number of adopted tomography models. More details for the calculation of vote maps can be found in ref(*15, 16*).

Here, high velocities are defined where the velocity perturbation is larger than the standard deviation of the corresponding model at given depth. Most models indicate the existence of a high-velocity anomaly penetrating the 660-km interface, which migrates westward as the depth increases. Therefore, both our observations and P-wave velocity models indicate a massive westward-dipping high-velocity anomaly at the bottom of the MTZ that is disconnected from the subducted Indian slab.

**Supplementary Figures**


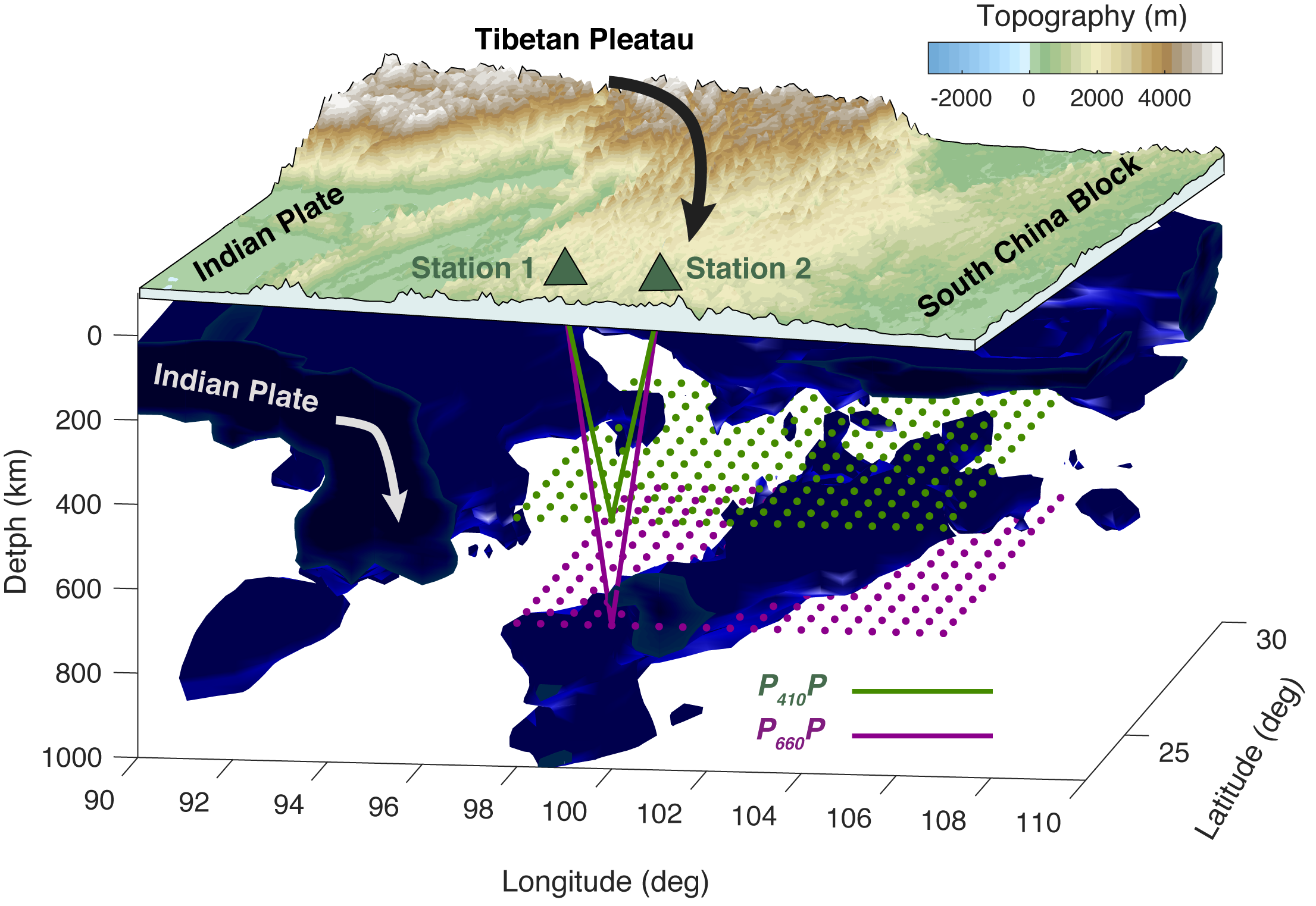


**Fig. S1.** Ray path geometries and the imaging region. Green and purple lines represent the ray paths of *P_410_P* and *P_660_P*, respectively. Green and purple dots denote the reflection points on the 410- and 660-km interfaces. The black arrow indicates the direction of continental extrusion. The white arrow indicates the direction of Indian plate subduction. Dark blue bodies represent the high-velocity (> 0.5%) anomalies calculated from the UU-P07 model (*14*).


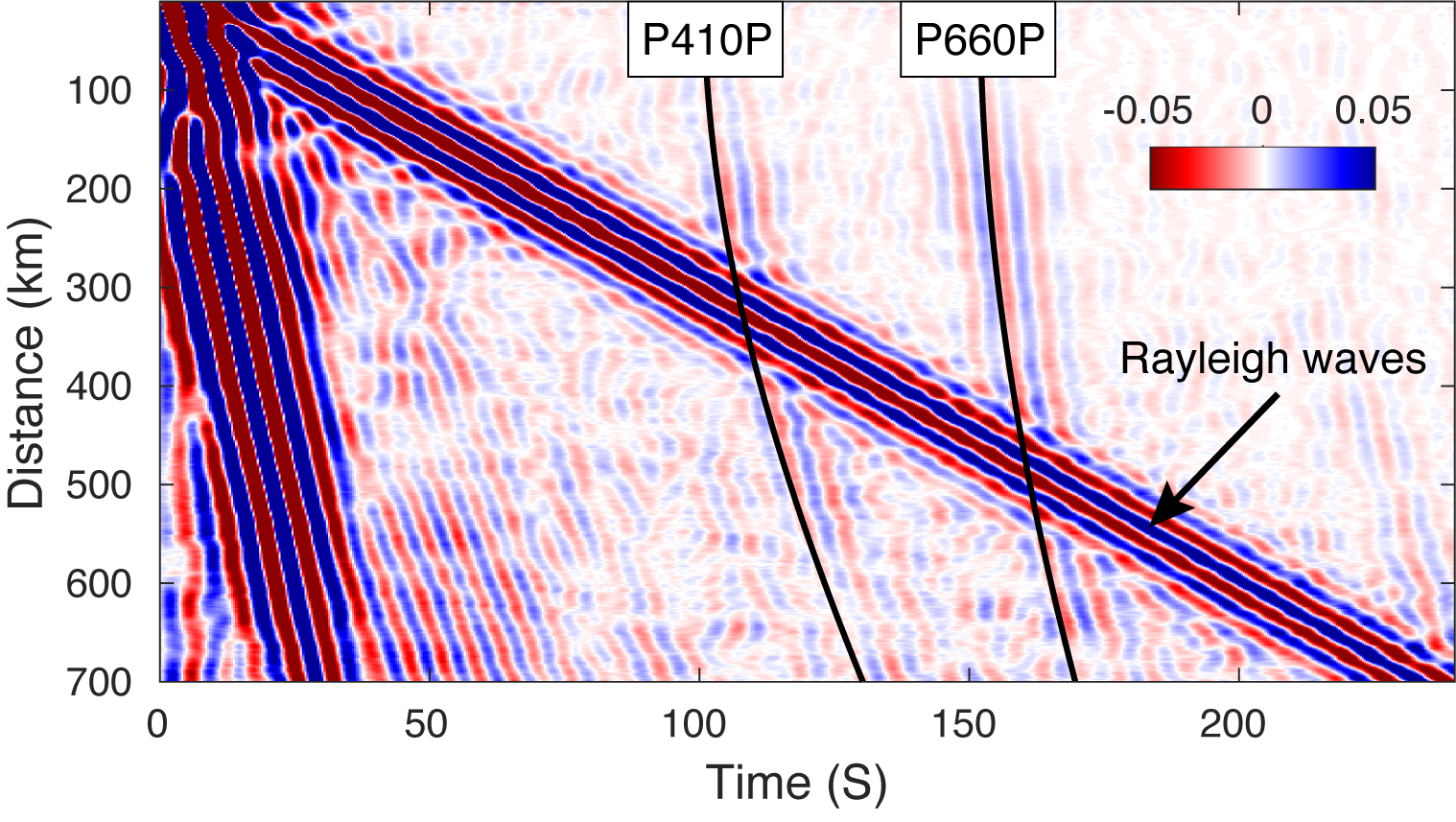


**Fig. S2.** Ambient noise cross-correlations arranged along distance. The background exhibits the NCFs, filtered to 0.1-0.2 Hz, aligned with respect to interstation distance. Black lines represent the theoretical traveltime curves of *P_410_P* and *P_660_P* calculated from the iasp91 model (*4*) with the TauP Toolkit (*5*).


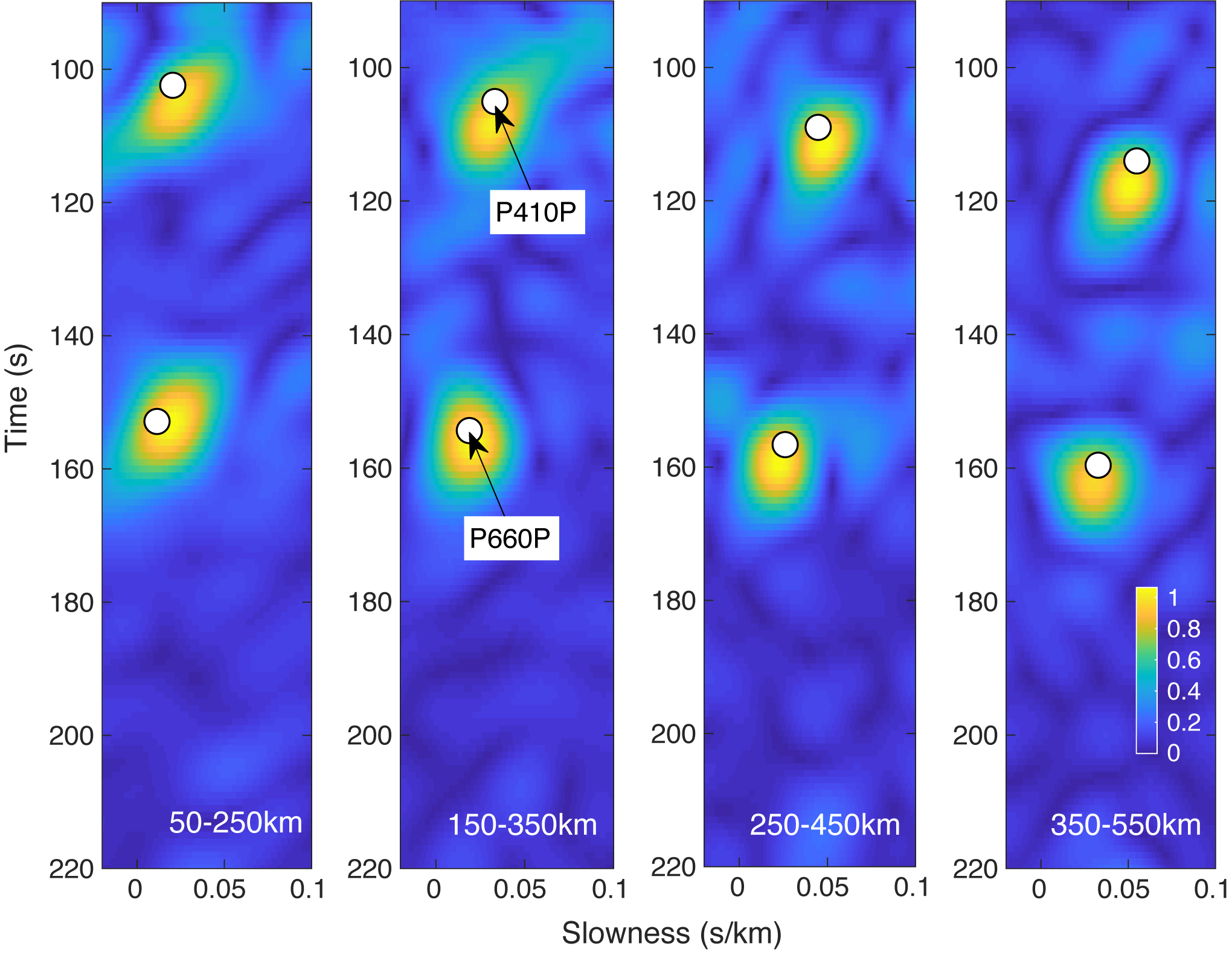


**Fig. S3.** Slant stacking of ambient noise cross-correlations. The colour of each panel shows the normalized envelope of slant stacking within four distance ranges (50-250 km, 150-350 km, 250-450 km, and 350-550 km). White circles denote the theoretical arrival times and ray parameters calculated with the TauP Toolkit (*5*), where the epicentral distance is set to the mean interstation distance of the ambient noise cross-correlations.


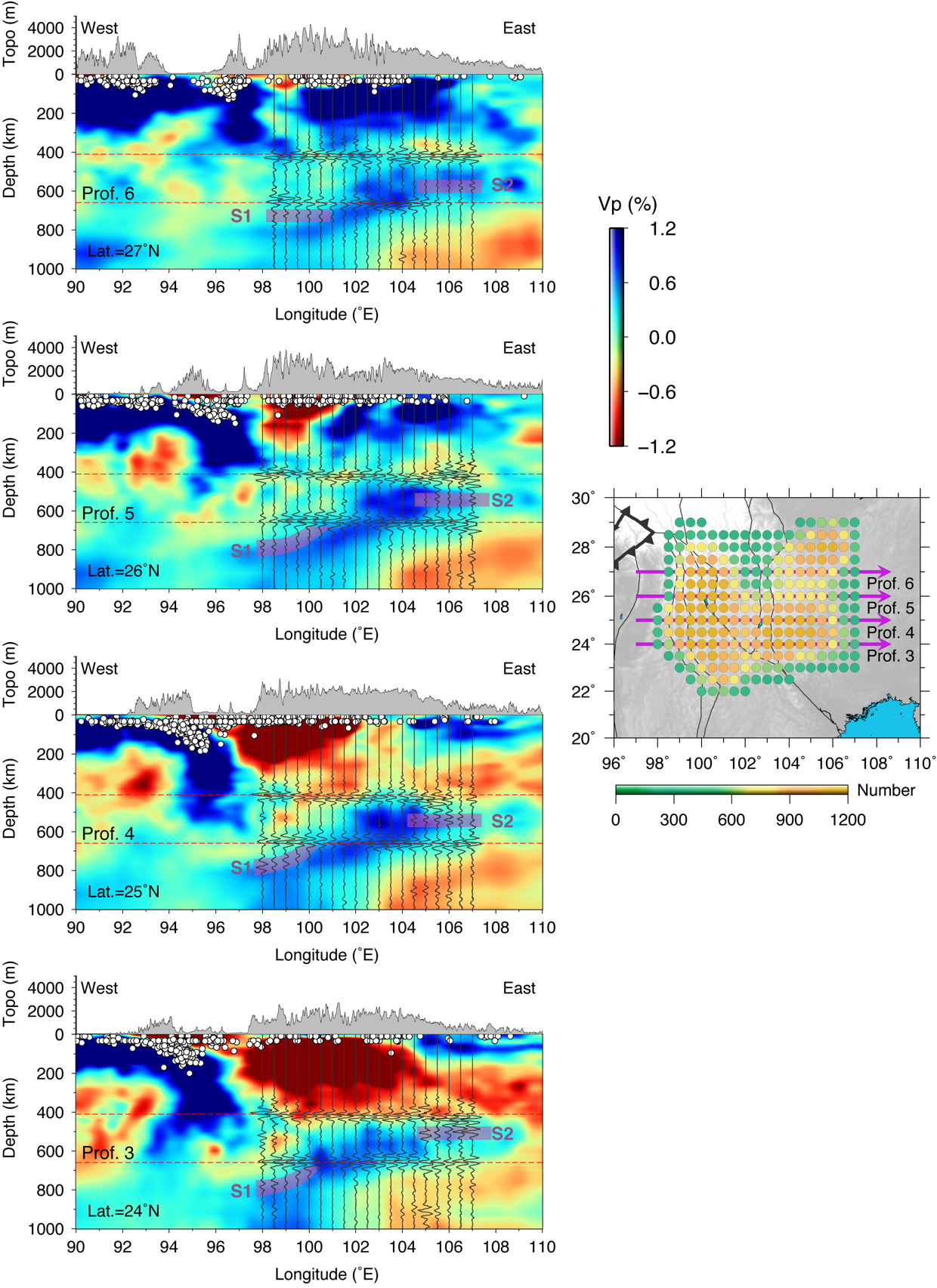


**Fig. S4.** Cross-sections of reflected waveforms and data density. The left four panels show the stacked waveforms along four profiles shown in the right panel as purple lines with arrows. The background shows the P-wave velocity perturbation calculated from the UU-P07 model (*14*). White circles present the seismicity (M>4) along each profile. The right panel shows the centres of common reflection stacking bins. The colour of the circles represents the number of stacked NCFs within each bin. Here, a bin is selected if the number of stacked NCFs is larger than 200.


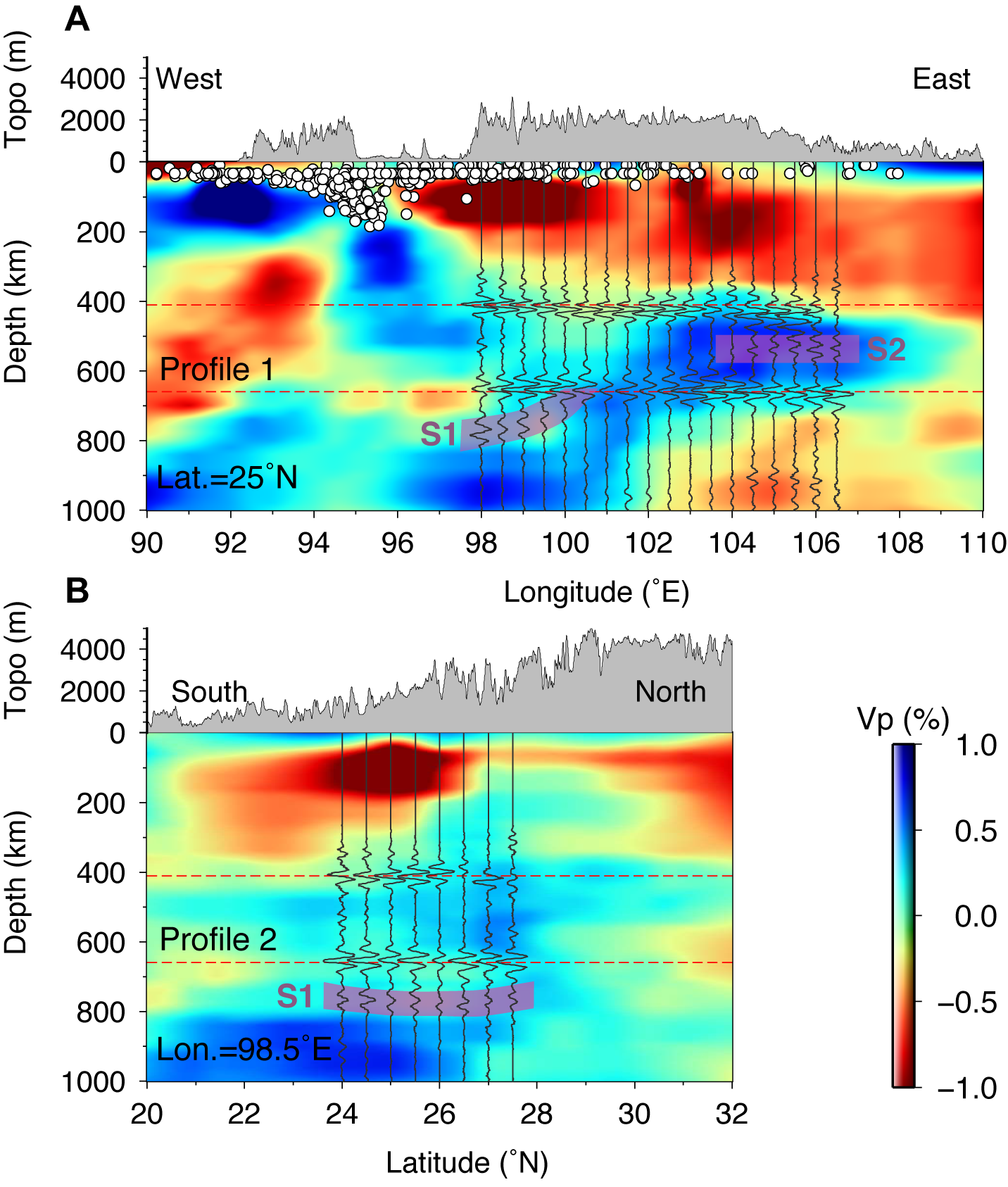


**Fig. S5.** Cross-sections of reflected waves and the MIT-P08 model. Here, Profile 1 and Profile 2 are the same as those in Fig. 3(**A** and **B**), but the background is extracted from the MIT-P08 model (*10*).


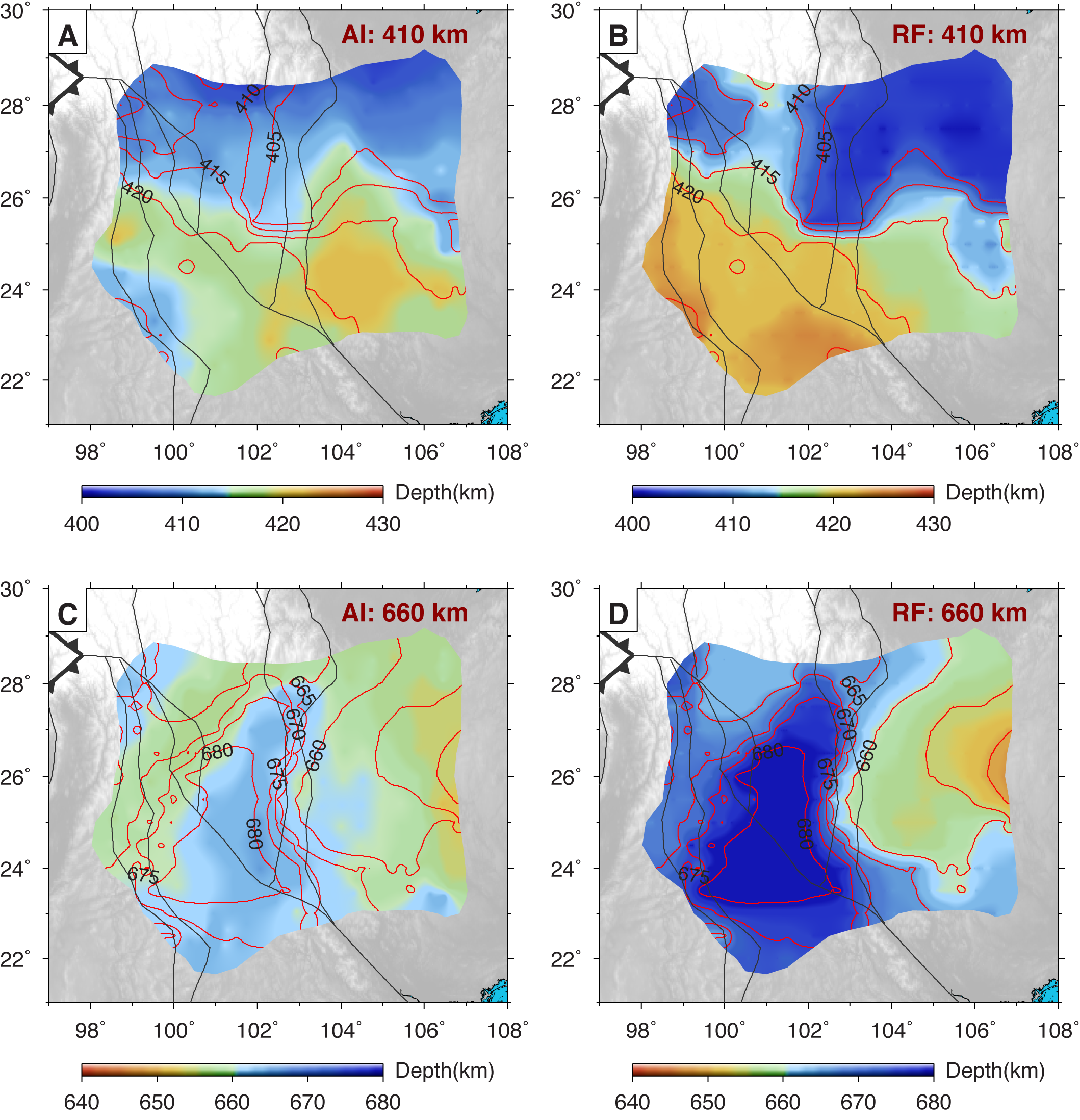


**Fig. S6.** Comparison among the topography of mantle transition zone discontinuities estimated from ambient noise interferometry (AI) and receiver function (RF), respectively. The red contours represent the mantle transition zone discontinuity depths estimated from RF. Here all the discontinuity tomography was calculated based the 1-D iasp91 model (*4*).


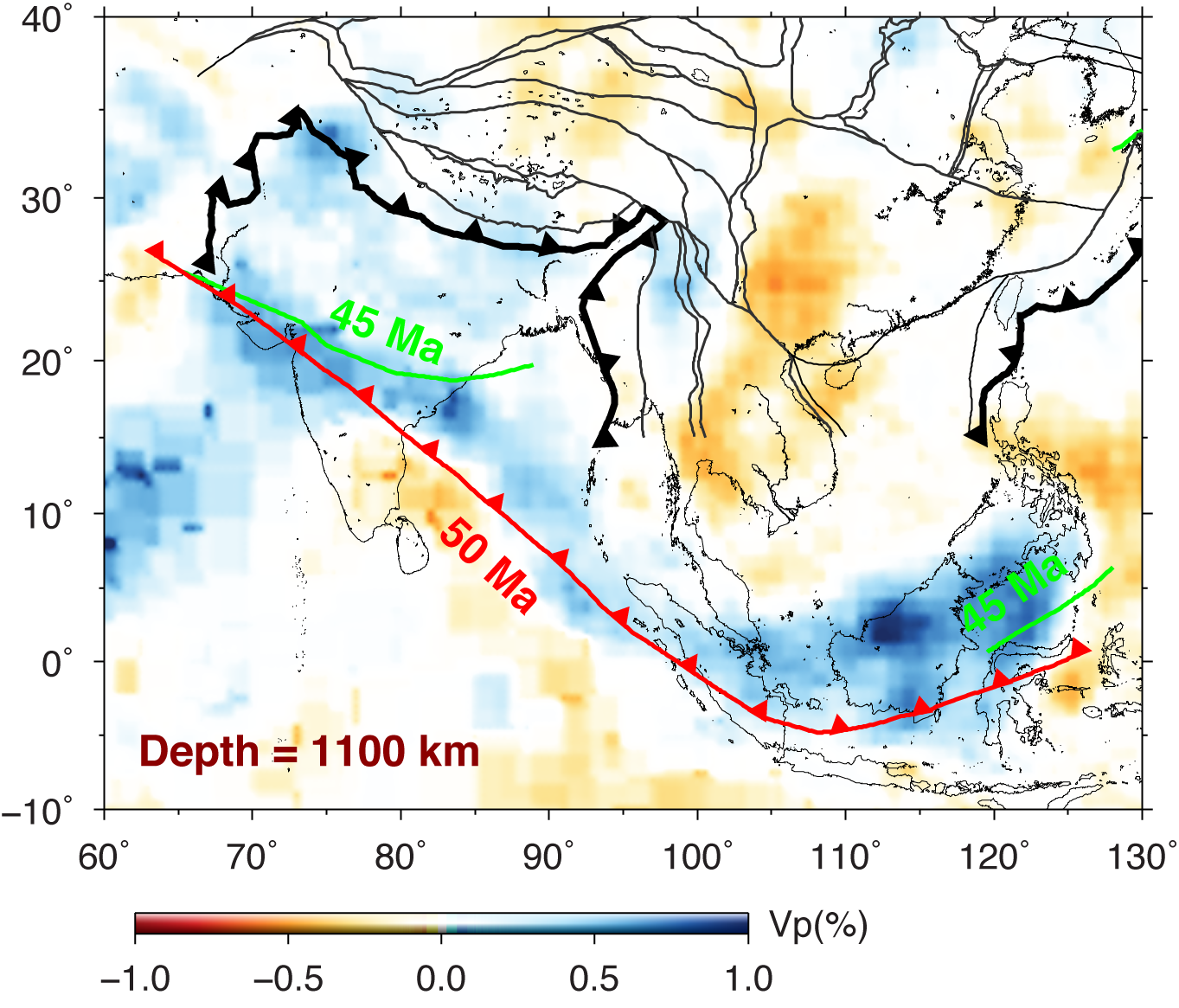


**Fig. S7.** The P-wave velocity perturbation at 1100 km extracted from the UU-P07 model (*14*). Thick black lines with triangles indicate the location of suture zones. Thin black lines show the major tectonic boundaries. Thick red and green lines present the location of reconstructed subduction zones 50 and 45 Ma, respectively. The location of subduction zones 50 and 45 Ma was estimated from ref(*17*) and supplementary data to ref(*18*), respectively. The high velocity along the reconstructed subduction zone potentially represent the submerged oceanic slab before the continental collision between India and Eurasia.


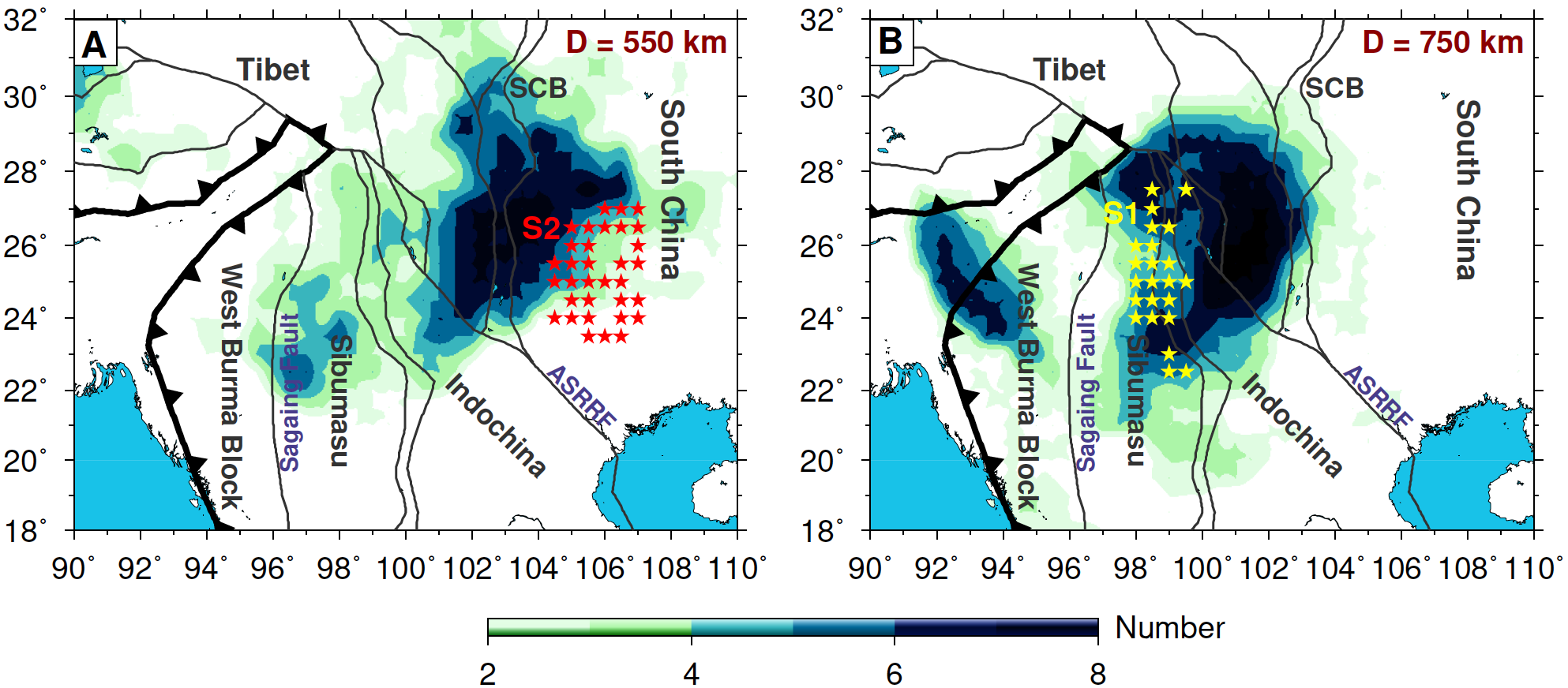


**Fig. S8.** High velocity vote maps around the 660-km interface. (**A**) The number of models identifying high velocities at 550 km among eight given models. The red stars show the location of S2 signals. (**B**) The same as (**A**) but at 750-km depth. The yellow stars indicate the location of S1 signals. Here, high velocity is defined when the velocity is larger than model’s standard deviation. Eight models (DETOX-P3 (*8*), GAP-P4 (*9*), Hosseini2016, MIT08 (*10*), MIT_USA_2011MAR (*11*), MIT_USA_2016MAY(*12*), TX2019slab-P (*13*), and UU-P07(*14*) provided by SubMachine (*15*)) were analyzed and the vote maps were generated following ref (*16*). The vote maps indicate a high-velocity anomaly around the 660-km interface, which migrates westward as the depth increases. SCB, Sichuan Basin; ASRRF, Ailao Shan-Red River Fault.


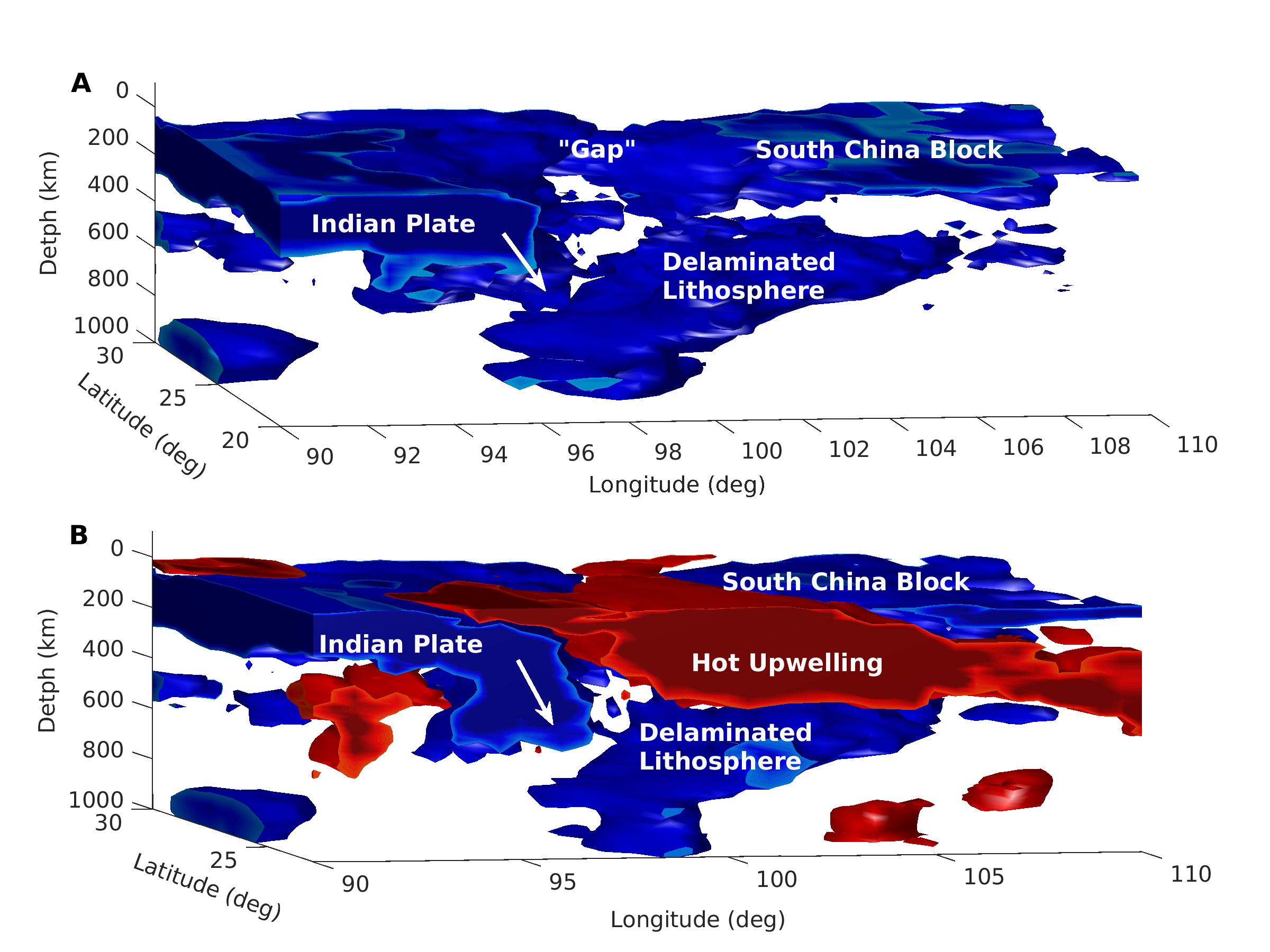


**Fig. S9.** Velocity anomalies calculated from the UU-P07 model. (**A**) The high-velocity anomalies (>0.5%). (**B**) Both the high-velocity anomalies (>0.5%) and low-velocity anomalies (<-0.6%).

**Supplementary References**

1. G. D. Bensen, M. H. Ritzwoller, M. P. Barmin, A. L. Levshin, F. Lin, M. P. Moschetti, N. M. Shapiro, Y. Yang, Processing seismic ambient noise data to obtain reliable broad-band surface wave dispersion measurements. *Geophys J Int* **169**, 1239-1260 (2007).

2. W. T. Wang, P. Gerstoft, B. S. Wang, Seasonality of P wave microseisms from NCF-based beamforming using ChinArray. *Geophys J Int* **213**, 1832-1848 (2018).

3. S. Rost, C. Thomas, Array seismology: Methods and applications. *Rev Geophys* **40**, (2002).

4. B. L. N. Kennett, E. R. Engdahl, Traveltimes for Global Earthquake Location and Phase Identification. *Geophys J Int* **105**, 429-465 (1991).

5. H. P. Crotwell, T. J. Owens, J. Ritsema, The TauP Toolkit: Flexible seismic travel-time and ray-path utilities. *Seismological Research Letters* **70**, 154-160 (1999).

6. M. Schimmel, H. Paulssen, Noise reduction and detection of weak, coherent signals through phase-weighted stacks. *Geophys J Int* **130**, 497-505 (1997).

7. J. Feng, H. Yao, P. Poli, L. Fang, Y. Wu, P. Zhang, Depth variations of 410 km and 660 km discontinuities in eastern North China Craton revealed by ambient noise interferometry. *Geophys Res Lett* **44**, 8328-8335 (2017).

8. K. Hosseini, K. Sigloch, M. Tsekhmistrenko, A. Zaheri, T. Nissen-Meyer, H. Igel, Global mantle structure from multifrequency tomography using P, PP and P-diffracted waves. *Geophys J Int* **220**, 96-141 (2020).

9. Y. Fukao, M. Obayashi, Subducted slabs stagnant above, penetrating through, and trapped below the 660 km discontinuity. *J Geophys Res-Sol Ea* **118**, 5920-5938 (2013).

10. C. Li, R. D. van der Hilst, E. R. Engdahl, S. Burdick, A new global model for P wave speed variations in Earth's mantle. *Geochem Geophy Geosy* **9**, (2008).

11. S. Burdick, R. D. van der Hilst, F. L. Vernon, V. Martynov, T. Cox, J. Eakins, G. H. Karasu, J. Tylell, L. Astiz, G. L. Pavlis, Model Update March 2011: Upper Mantle Heterogeneity beneath North America from Traveltime Tomography with Global and USArray Transportable Array Data (vol 83, pg 23, 2012). *Seismological Research Letters* **83**, 280-280 (2012).

12. S. Burdick, F. L. Vernon, V. Martynov, J. Eakins, T. Cox, J. Tytell, T. Mulder, M. C. White, L. Astiz, G. L. Pavlis, R. D. van der Hilst, Model Update May 2016: Upper-Mantle Heterogeneity beneath North America from Travel-Time Tomography with Global and USArray Data. *Seismological Research Letters* **88**, 319-325 (2017).

13. C. Lu, S. P. Grand, H. Y. Lai, E. J. Garnero, TX2019slab: A New P and S Tomography Model Incorporating Subducting Slabs. *J Geophys Res-Sol Ea* **124**, 11549-11567 (2019).

14. R. Hall, W. Spakman, Mantle structure and tectonic history of SE Asia. *Tectonophysics* **658**, 14-45 (2015).

15. K. Hosseini, K. J. Matthews, K. Sigloch, G. E. Shephard, M. Domeier, M. Tsekhmistrenko, SubMachine: Web-Based Tools for Exploring Seismic Tomography and Other Models of Earth's Deep Interior. *Geochem Geophy Geosy* **19**, 1464-1483 (2018).

16. G. E. Shephard, K. J. Matthews, K. Hosseini, M. Domeier, On the consistency of seismically imaged lower mantle slabs. *Sci Rep-Uk* **7**, (2017).

17. A. Replumaz, H. Karason, R. D. van der Hilst, J. Besse, P. Tapponnier, 4-D evolution of SE Asia's mantle from geological reconstructions and seismic tomography. *Earth Planet Sc Lett* **221**, 103-115 (2004).

18. S. Zahirovic, K. J. Matthews, N. Flament, R. D. Muller, K. C. Hill, M. Seton, M. Gurnis, Tectonic evolution and deep mantle structure of the eastern Tethys since the latest Jurassic. *Earth-Sci Rev* **162**, 293-337 (2016).
